# Characterizing the Dynamic Taste and Retro-Nasal Aroma Properties of Oral Nutritional Supplements Using Temporal Dominance of Sensation and Temporal Check-All-That-Apply Methods

**DOI:** 10.3390/foods9101456

**Published:** 2020-10-13

**Authors:** Thomas Delompré, Louis Lenoir, Christophe Martin, Loïc Briand, Christian Salles

**Affiliations:** CSGA (Centre des Sciences du Goût et de l’Alimentation), AgroSup Dijon, CNRS, INRAE, Université de Bourgogne-Franche Comté, 21000 Dijon, France; thomas.delompre@inrae.fr (T.D.); lenoir0louis@gmail.com (L.L.); christophe.martin@inrae.fr (C.M.); loic.briand@inrae.fr (L.B.)

**Keywords:** orodispersible nutritional supplements (ODTs), temporal dominance of sensations (TDS), temporal check-all-that-apply (TCATA), dynamic perception, flavoring impact

## Abstract

Orally Disintegrating Tablets (ODTs) are used to restore the nutritional status of people suffering from swallowing pathologies such as dysphagia. ODTs are consumed without water intake and are easily swallowed. The main active compounds of ODTs are vitamins and minerals. These nutrients can have a bad taste or aftertaste, which can be masked by sweetening or flavoring. To ensure the effectiveness of masking strategies and to prescribe a product to patients with acceptable sensory qualities, it is essential to perform a precise and complete sensory characterization of these ODTs. Temporal dominance of sensations (TDS) and temporal check-all-that-apply (TCATA) methods were chosen to characterize the temporal sensory perceptions during the consumption of four ODTs varying in galenic forms and flavoring because of their particularly acute temporality in flavor perception. The ODTs presented common and individual sensory properties, mainly related to their galenic form and to the nature of the active ingredients. The use of a nose-clip to stop retro-nasal airflow showed that flavoring had a minimal impact on the sensory taste qualities of the ODTs. A comparison between the TDS and TCATA results indicates that these tests are capable of providing complementary information on the dynamic sensory qualities of the products studied. Although results were generally similar for both methods, TDS showed a greater number of differences in sensory taste attribute, whereas TCATA was more discriminating. These methods allowed for a better understanding of the evolution of sensory perceptions of these ODTs during their consumption, which could help to optimize masking strategies and develop new products with acceptable sensory properties.

## 1. Introduction

Nutrients such as vitamins and minerals are essential for correct body function. As nutrients are mainly provided by a healthy and balanced diet, nutrient deficiencies can appear during certain pathologies such as cancers or gastrointestinal disease [1,2]. Nutritional supplements are used to rectify these nutrient deficiencies and to maintain nutritional status [3,4]. These nutritional supplements are a concentrated source of nutrients with nutritional and/or physiological effects [5]. The oral route is the preferred route of nutritional supplement administration for solid forms such as tablets or capsules. Solid forms of nutritional supplements have started gaining in popularity as drug delivery systems because they are easy to administer, allow exact dosages of the active compounds, and facilitate patient compliance [6]. However, approximately 35 to 50% of the elderly and young population suffer from swallowing pathologies such as dysphagia [7]. Those suffering from dysphagia have difficulty swallowing prescribed capsules or tablets. These swallowing disorders may lead to a significant decline in patient compliance and reduce the effectiveness of treatment [6]. Therefore, pharmaceutical companies have developed new solid forms called orodispersible tablets or orally disintegrating tablets (ODTs) [8]. These ODTs combine the advantages of solid and liquid forms. These tablets disintegrate in the mouth within three minutes of when they come into contact with saliva, mitigating water intake and swallowing difficulty for the patient [9]. Today, ODTs represent a large proportion of nutritional supplements available on the pharmaceutical market and are the preferred forms for oral nutritional supplement administration. Many nutrients present in these ODTs such as vitamins, minerals, or certain amino acids are known to be at the origin of negative perceptual sensations such as bitterness or astringency [10]. As ODTs disintegrate in a patient’s mouth, these active compounds are in direct contact with the taste detection system and often exhibit bad taste such as bitterness and an unpleasant aftertaste. Thus, this excludes substitution as a strategy to avoid off tastes. Therefore, it is essential for manufacturers to perform effective masking strategies such as the inclusion of flavoring or sweetening products [11]. To ensure the effectiveness of these masking strategies and to sell products with acceptable sensory qualities, it is essential to perform sensory analysis to precisely characterize the sensory qualities of ODTs. Once these ODTs are put in the mouth, tastants and odorants will generally be released for a longer time than when eating foods [9]. This dynamic release plays an important role in the sensory perception of ODTs and in their appreciation by the consumer. Two sensory analytical methods have been developed to analyze these dynamic perceptions: temporal dominance of sensations (TDS) and temporal check-all-that-apply (TCATA). The TDS method identifies the sensation perceived as “dominant” at a specific moment of product evaluation [12]. TCATA captures all sensations perceived at each moment during oral processing [13]. These two analytical methods present sensory protocols and similar data processing procedures. These methods have major advantages such as reduced panelist training and the simultaneous use of ten descriptors over other methods of temporal analysis, which allow for a comprehensive characterization of the product studied [14,15,16]. TDS and TCATA have been used to examine the flavor or texture of many liquid, semi-solid or solid products [17,18,19,20,21,22,23]. To date, no study has referred to the use of these two sensory analytical methods to characterize the sensory qualities of ODTs, which have a real perceptual temporality during oral processing.

The purpose of the current research was to study the dynamics of perception of the taste and retro-nasal odor of ODTs to better control and limit their off flavors. Two temporal sensory analytical methods were used to study the sensory qualities of these products with particular characteristics. We assumed that the use of certain aromas contributed to increasing taste perception with negative valences such as bitterness; therefore, the contribution of aroma to savoring perception was evaluated. Although widely used with products other than ODTs, we determined whether these temporal analytical methods provided accurate and similar sensory information for these ODTs considering the dynamic aspect of sensory perception [24,25,26,27,28]. Moreover, neither TDS nor TCATA has been used for temporal perception studies of ODTs, and we found it relevant to test and compare these two methods for the temporal perception study of such products with particular temporal flavor properties.

## 2. Materials and Methods

### 2.1. Stimuli

Four ODTs, available in drugstores, of various galenic and aromatized forms were evaluated: two ODTs in the form of powders called sticks due to their cylindrical packaging, ODT_exotic and ODT_cola, and two ODTs in the form of tablets called fizzy tabs due to the tingling sensation felt on the tongue when the tablet disintegrates in the mouth, ODT_red and ODT_citrus. These ODTs are comprised of different active ingredients such as vitamins and minerals as well as sweeteners and many excipients including flavor compounds (Table 1). The ODTs were provided by BAYER SAS (Global innovation Center, Gaillard, France). The samples were stored at 20 °C in a ventilated darkroom until use.

### 2.2. Panelists

Fifteen panelists (22–67 years old, four males and 11 females) were recruited from Dijon (France) and surrounding areas. The selection was based on their availability, their interest in the study, and their sensory ability such as taste and olfactory capacity (sensitivity and recognition tests). All panelists were naïve to classical descriptive sensory analytical techniques such as quantitative descriptive analysis profiles and descriptive temporal analytical methods [29,30]. An ethical committee approved this study (Personal Protection Committee 2018-A01342-53). All panelists signed an informed consent form before the study. The panelists were asked to not drink, eat, or use perfume one hour before sensory analysis. The study was carried out over three months.

### 2.3. Attributes

Based on the results obtained in a previous sensory profile on similar nutritional supplements (data not shown), 10 attributes were selected based on their relatively high perception intensity, discriminating power, and relevance to provide an answer to the objective of the study [14,31,32]. To compare the results obtained, the same attribute list was used for TDS and TCATA. ODT oral processing was approximately one minute, and the order of the attributes was randomized for each of the panelists to avoid selecting attributes based on their position in the list.

### 2.4. TDS (Temporal Dominance of Sensations)/TCATA (Temporal Check-All-That-Apply) Sensory Protocol

Organization of the sessions: The panelists were randomly divided into two groups. The first group started with TDS and then moved to the TCATA session, and the other group started with TCATA followed by the TDS sessions. All panelists performed four repetitions of each test under two conditions: with nose clips (WNC) and without nose clips (WoNC).

Training sessions: Before performing the measurement sessions, two training sessions were provided in the same week to familiarize the panelists with the attribute list, sensory tests used, and the signal acquisition software. The first training session was devoted to the presentation of the two dynamic sensory tests, irrespective of the method by which the panelists would start. The temporal and dynamic aspects of a tasting was debated and exemplified. For the TDS, the concept of “dominant attribute” was defined as an attribute that attracts the most attention, but is not the most intense [15,16]. For the TCATA, the concept of “applicable attributes” was also explained and exemplified [13]. All panelists were informed that a single descriptor could not be selected at any given time during the tasting for the TDS, while several attributes could be selected for the TCATA. It was also clarified to all panelists that for the TCATA, the attributes applicable to the products during the tasting should be checked and the attributes that are no longer applicable should be unchecked. It was clearly stated to the panel that there were no restrictions on the selection of the number of attributes and that the same attributes could be re-selected if perceived again. Sensory references were presented to panelists to define each of the attributes (Table 2). During the second training session, an evaluation session was performed on two similar products to those included in the study. The objective of this session was to familiarize the panelist with the signal acquisition software and marking instructions for both methods. This training was performed to ensure that all panelists understood the methodology to be followed and the instructions to be applied. The marking sessions were conducted in individual boxes equipped with a computer, and the data were collected using the Internet software TimeSens V1.

Measure sessions: Four samples, randomly coded by three-digit codes and placed in white cups, were distributed to each panelist. The instructions were to consume the whole sample (0.85 ± 0.05 g for powder forms and 0.72 ± 0.05 g for tablet forms; this amount represents half of the ODTs) were given to all panelists. Since manufacture recommendations recommend an oral dose of two ODTs per day, four products could be tested during the same sensory analysis sessions. The order of product presentation was balanced for each repetition and for all panelists using Williams Latin Squares. The panelists were asked to rinse their mouths with pure water and wait a minimum of two minutes between each sample. To evaluate the impact of aromatization on taste perceptions, the evaluations were also performed with a nose-clip (WNC) by each panelist for each sensory method and for each product, according to the protocol established previously. A total number of eighteen sessions was conducted including training and measure sessions.

### 2.5. Data Analysis

Data collection and analysis were similarly performed for both tests using the statistical models available in TimeSens. TDS and TCATA data were acquired by the software every 250 ms. First, the product curves for the TDS and TCATA methods were constructed by plotting the dominance (TDS) and citation frequency (TCATA) of each attribute by standardized time [13,33]. Time standardization was applied to homogenize the panelists oral flavor duration (OFD). Data of each subject and each product were standardized according to individual OFD. After standardization, the *X*-axis of the TDS and TCATA curves did not represent the real time of the OFD, but the period from first scoring (x = 0) to last scoring (x = 1) [34]. The TDS curves contained two additional lines allowing for statistical interpretation. A “chance line”, which corresponded to the probability of selecting an attribute at random, was set in this study at 0.1 (1/10 attributes). A second line, which represented the significance threshold (95% confidence) for each attribute, called the “significance line”, was fixed as 0.16 [33]. An attribute was qualified as significant if it had a proportion of selection greater than this significance line for a given time period.

An overview of the dynamic sensory characteristics of the studied products was observed through a product map generated by performing canonical variate analysis (CVA) on the perception duration of each attribute of the sample by the panelists’ means evaluation. The product map was generated by using a statistical test available in TimeSens (CVApack R-package) applied to data from TDS and TCATA [35]. In this multivariate analysis, products were identified as fixed effects and panelists as the random effects. The result of this statistical test was a product map with 90% confidence ellipses for each sample, which corresponded to the marking of each subject. The layout of ellipses and segment lines connecting samples provided information about the degree of similarities and differences between samples.

A trajectory map representing the oral disintegration path of each sample was established by performing principal component analysis (PCA) on the standardized data of TDS and TCATA (tempR package of TimeSens) [34]. This graph highlights the sensory perception evolution of product ingestion until the end of the evaluation. This graph was constructed from a given table, and the lines represent the dominance rate or the proportion of citation (depending on the method studied) of each product at 11 time points (i.e., 0%, 10%, 20%, and so on up to 100%), and the attributes tested are in the columns [36].

To examine the impact of the galenic form on the sensory perception duration in the mouth, oral flavor duration (in seconds) was determined, and ANOVA with repeated measurements that included the main effect of the product was performed (model: OFD ~ product + err) [37]. Tukey’s honest significant difference (HSD) with a level of significance of *p* < 0.05 was performed to check the samples that differed significantly from one another.

To investigate the flavoring effect on dynamic taste perceptions, difference curves between a given pair of products (evaluation with nose-clip and evaluation without nose-clip), based on a Fisher test (95% significance), were generated using XLSTAT 2020.2.3 software. A no-displayed curve corresponding to an attribute for a given time period meant that no difference could be observed between samples for this attribute. One-way ANOVAs (tempR package) that included the main effect of the evaluation condition (with nose-clip and without nose-clip) were separately performed on TDS and TCATA data to study the flavoring effect on dominance and citation duration of all attributes (model: dominance or citation duration ~ product + err). The dependent variables were the dominance duration (TDS) and the citation proportion (TCATA) of all attributes for each product. Post-hoc Tukey’s honest significant difference (HSD) test with a significance level of *p* < 0.05 was performed to find means that were significantly different from each other.

## 3. Results

### 3.1. Dynamic Product Curves

Dynamic product curves were generated for both sensory tests to perform a product sensory characterization and to evaluate the evolution of their sensory qualities during tasting. A comparison, although exclusively visual, was carried out to establish similarities or differences between these two dynamic sensory methods. These curves were generated in a standardized format, where the x-axis represents the percentage of oral treatment time and the Y-axis represents the dominance rate in TDS and the citation proportion in TCATA.

#### 3.1.1. TDS Curves

**Figure 1:**
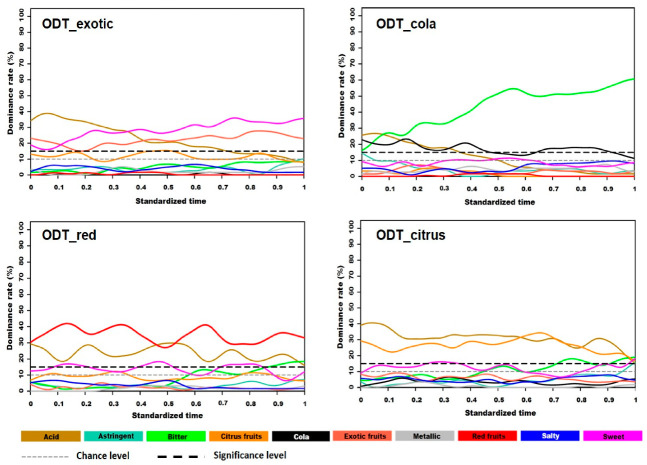
Standardized TDS (Temporal Dominance of Sensations) curves of four ODTs (Orally Disintegrating Tablets).

The TDS curves are presented in Figure 1. Since the aroma compounds follow the retro-nasal route to reach receptors in the nasal olfactive mucosa, the corresponding sensory attributes were significantly rated throughout the tasting of ODT_exotic, ODT_red, and ODT_citrus with a dominance rate between 30% and 40%. Cola, which was the aroma used in ODT_cola, was the only attribute that was not significantly dominant throughout the oral flavor duration (OFD). Concerning the taste attributes, acid was perceived as dominant in the first third of the tasting for all four products. The dominance rate of this attribute gradually decreased and became non-significant in the final third of the OFD for ODT_exotic and ODT_cola, unlike ODT_red and ODT_citrus. ODT_exotic was characterized by the dominance of a sweet taste, which gradually increased to reach a dominance rate of 35% at 70% of the mastication time. For ODT_cola, a bitter taste was the only significantly dominant attribute throughout the OFD, with a dominance rate between 50% and 60% in the final half. ODT_red and ODT_citrus had relatively similar dynamic sensory profiles. The large dominance of acid was observed from the start until the end of the evaluation. Sweet and bitter were also perceived as dominant, but only at very low dominance rates, remaining close to the significance limit. For the two products, bitter dominance became significant at the end of OFD, while the dominance of acid fell below the significance threshold. ODT_citrus differed from ODT_red with a more marked dominance of a bitter taste in the last quarter of the OFD that remained until the end. A sweet taste was also dominant at several times in the temporal sensory profile of ODT_red (10–20%, 40–50%, 70–85%), unlike ODT_citrus (25–35%).

#### 3.1.2. TCATA Curves

**Figure 2:**
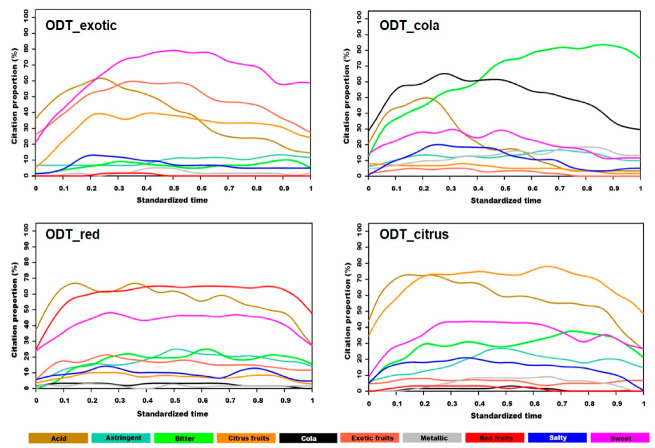
Standardized TCATA (Temporal Check-All-That-Apply) curves of four ODTs (Orally Disintegrating Tablets).

The dynamic TCATA curves for the four products are shown in Figure 2. TCATA results are comparable to those obtained by TDS with a few exceptions. Citation proportions of exotic fruits and cola, which were characteristic attributes of the aromas used in ODT_exotic and ODT_cola, respectively, gradually decreased in the second half of the OFD, reaching 30% at the end of the evaluation. The gradual decrease in aroma attribute citation was not observed for red fruit and citrus fruit attributes in ODT_red and ODT_citrus, respectively, for which the TCATA curves exhibited a plateau surrounded by rapid increasing and decreasing phases. For both products, the citation proportions of the main taste attributes, acid, sweet, and bitter were different. In particular, acid was mostly present in the initial one-third of the mastication time. For ODT_exotic and ODT_cola, a strong decrease in acid citation proportion was observed after 25% of the OFD. With a high citation proportion (80%), mainly in the final two-thirds OFD, a sweet taste strongly characterized ODT_exotic. A sweet taste was also found in the other evaluated samples, but with lower and relatively stable citation proportions: between 15 and 30% for ODT_cola and between 20 and 40% for ODT_red and ODT_citrus. Bitter citation proportions were high in the final two-thirds of the mastication time for ODT_cola and differed from those of ODT_red and ODT_citrus, which also exhibited different aromatic attributes, as reported above. Indeed, the citation proportion of this attribute was higher on average by 10% for ODT_citrus than for ODT_red from the beginning to the end of tasting. Astringent, with a 20% citation proportion in the final two-thirds of the OFD, was a common attribute for ODT_red and ODT_citrus.

### 3.2. Product Map

An overview of the dynamic sensory characteristics of the studied products was observed through a product map generated by performing CVA on the perception duration of each attribute of the sample by the panelists’ means evaluation. This graphical representation allowed us to determine whether the products had been differentiated from each other by the dominance durations of each attribute. The proximity degree of the ellipses provided information on the similarity degree between products. A vector that indicated the direction and whose length was proportional to the product discrimination provided by this attribute represented each attribute. The F-statistic gave information regarding product discrimination and allowed for a comparison of the discriminating power of both methods.

**Figure 3:**
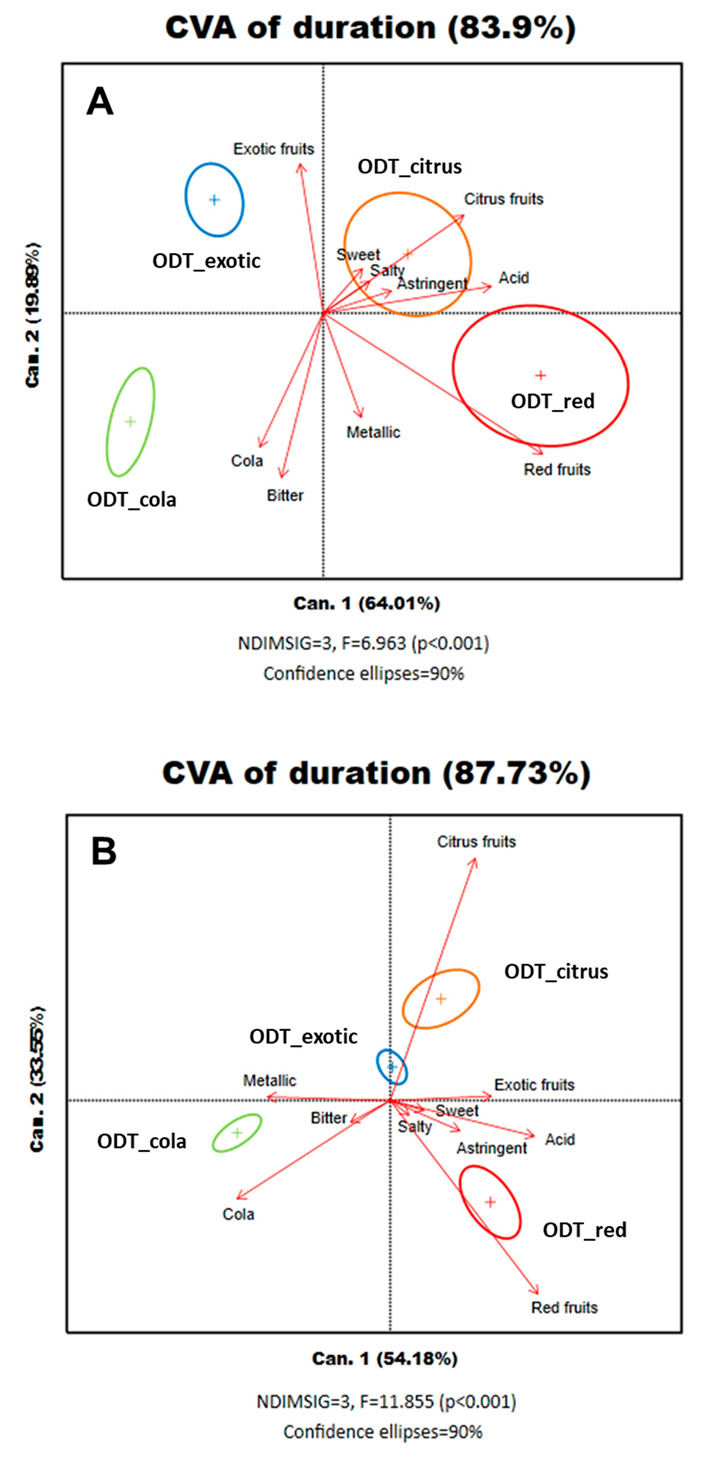
(**A**) TDS (Temporal Dominance of Sensations) product map discriminating the four products using CVA (Canonical Variate Analysis). Colored ellipses represent the products: blue, ODT_exotic; green, ODT_cola; red, ODT_red; and orange, ODT_citrus. The attribute names are in black, and attributes are represented by red vectors. (**B**) TCATA (Temporal Check-All-That-Apply) product map discriminating the four products using CVA analysis. Color ellipses represent the products: blue, ODT_exotic; green, ODT_cola; red, ODT_red; and orange, ODT_citrus. The attribute names are in black, and attributes are represented by red vectors.

As shown in Figure 3, the products were significantly discriminated with a *p* < 0.001 and an F-statistic of 6.96 for TDS (Figure 3A) and an F-statistic of 11.85 for TCATA (Figure 3B). The first dimension of the biplot explains, for both tests, a significant amount of variability between the products (i.e., 83.90% for TDS and 87.73% for TCATA). Considering the taste sensory attributes as a whole, only bitter distinguished one product from the three others. In both product maps, exotic fruits-citrus fruits characterized ODT_exotic and ODT_citrus, ODT_cola had cola–bitter characteristics, and ODT_red was marked by red fruit traits. Although present in all products studied, the attributes metallic, sweet, astringent, and salty had short vectors. In other words, these attributes did not play a significant role in discriminating samples.

### 3.3. Sensory Trajectory

**Figure 4:**
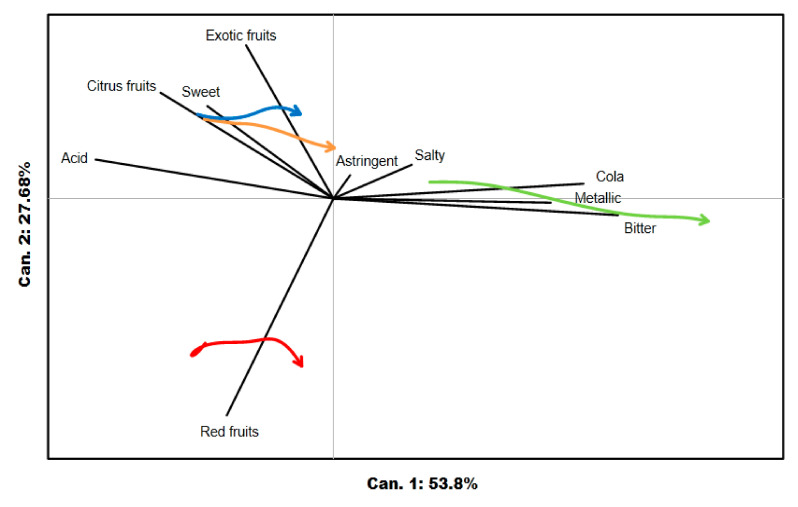
The sensory trajectories of the four products are represented on a PCA (Principal Component Analysis) biplot for TDS (Temporal Dominance of Sensations), at 10 equally spaced time points during oral processing. The sensory trajectories of the four products are each indicated by a different color: blue, ODT_exotic; green, ODT_cola; red, ODT_red; and orange, ODT_citrus.

The sensory trajectories of the dominance rates for the TDS data are plotted in a PCA biplot (Figure 4). The two dimensions explain 81.48% of the variance. The first dimension, which explains 53.80% of the variance, was guided strongly by bitter, acid, cola, and metallic attributes. The aromatic attributes of red fruits and exotic fruits strongly influenced the second dimension. All samples had acid as the first perceived dominant attribute and bitter as the last perceived dominant attribute, which explained their trajectories from left to right in the multidimensional plane. The sensory trajectories for the four samples can be classified into three groups based on their initial perception and the trajectory to reach the last perceived attribute. ODT_exotic and ODT_citrus, with close flavoring, had relatively similar sensory trajectories. These products started from an acidic perception and moved toward exotic fruits for ODT_exotic and citrus fruits for ODT_citrus, then became sweet, which was more marked for ODT_exotic, and ended at bitter. The ODT_cola trajectory began at the acid attribute and then moved to the cola aromatic attribute to finish at the bitter attribute. In turn, ODT_red followed the attribute path of acid to red fruits to bitter.

**Figure 5:**
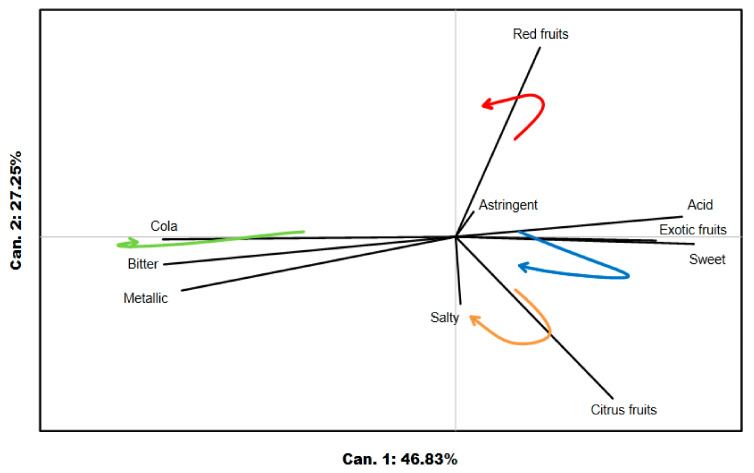
The sensory trajectories of the four products are represented on a PCA (Principal Component Analysis) biplot for TCATA (Temporal Check-All-That-Apply) at 10 equally spaced time points during oral processing. The sensory trajectories of the four products are each indicated by a different color: blue, ODT_exotic; green, ODT_cola; red, ODT_red; and orange, ODT_citrus.

The observation of the PCA biplot generated with the TCATA data indicated that the first two dimensions explained 74.08% of the variance (Figure 5). Although attribute positions were different in the multidimensional map, the interpretation was the same as that provided for TDS. All the samples began their sensory trajectory at the acid attributed and ended at the bitter attribute. However, due to the positioning of the exotic fruit and sweet attributes on the first dimension and the citrus fruit attribute on the second dimension, the distinction between ODT_exotic and ODT_citrus was more marked for TCATA than for TDS.

### 3.4. Oral Flavor Duration

The oral flavor duration (OFD), which corresponds to the perception duration of the different attributes in the mouth, was analyzed to determine whether the dosage form used had an effect on the process time of the products. Significant differences were found between the samples for TDS and TCATA, with an F-statistic of 30.78 (*p* < 0.001) and an F-statistic of 28.69 (*p* < 0.001), respectively (Figure S1). For TDS, the OFD for ODTs in tablet form was on average 84.44 s (ODT_citrus) and 94.09 s (ODT_red), while for the ODTs in powder form, the OFD was approximately 47.55 s (ODT_exotic) and 51.28 s (ODT_cola) (Figure S1A). The results obtained by TCATA were similar, with differences of a few seconds compared to those obtained by TDS (Figure S1B). These small differences in time were probably due to the nature of the two tests, which have different rating guidelines.

### 3.5. Impact of Flavoring on Taste Sensory Perception

Evaluation of the four products was also conducted under a pinched-nose condition (i.e., the with nose-clip (WNC) groups). Obstructing airflow between the nasal and oral cavities minimizes the effect of aromas on dynamic sensory perceptions. Thus, by comparison with open-nose conditions, it was possible to evaluate the impact of flavoring on dominance (TDS) or citation rates (TCATA) and on the dominance or citation duration of taste attributes, considering that the aromas were tasteless at the used concentration.

#### 3.5.1. Difference Curves

**Figure 6:**
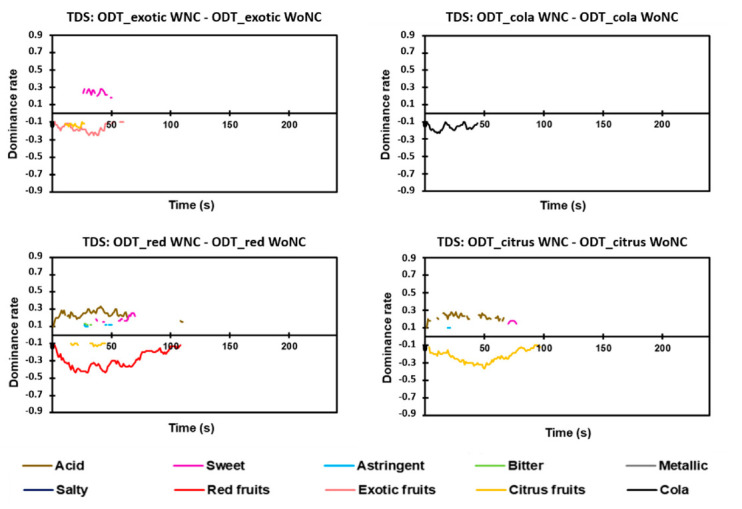
Difference curves for TDS (Temporal Dominance of Sensations) between a given pair of the same product (nose-clip evaluation and without nose-clip evaluation) based on 95% Fisher’s exact test. WNC, with nose-clip. WoNC, without nose-clip.

**Figure 7:**
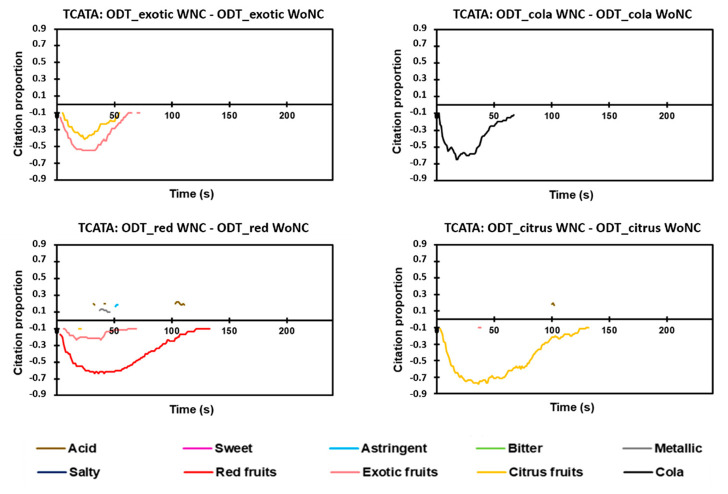
Difference curves for TCATA (Temporal Check-All-That-Apply) between a given pair of the same product (nose-clip evaluation and without nose-clip evaluation) based on 95% Fisher’s exact test. WNC, with nose-clip. WoNC, without nose-clip.

The difference curves show how flavoring can cause differences in the dynamic perception of the taste attributes. The difference curves obtained by difference between the products evaluated with a nose-clip (WNC) and without a nose-clip (WoNC) are presented in Figure 6 for TDS and in Figure 7 for TCATA. For TDS, the suppression of flavoring perception of ODT_red and ODT_citrus was associated with an increase in the dominance rate of acid in the initial two-thirds of the OFD (Figure 6). Slight variations in the dominance rates of sweet and astringent were also observed. The ODT_exotic difference curves revealed a higher dominance rate of the sweet attribute in WNC than in WoNC, mainly in the last third of the OFD. No difference in the dynamic perception of taste qualities was observed for ODT_cola.

The TCATA difference curves showed some discrepancies in comparison to the TDS differences curves. No difference was observable in taste attributes for ODT_exotic, ODT_cola, and ODT_citrus. Only metallic and acid had a higher proportion of citations at particular times of the OFD for ODT_red in the WNC than in the WoNC group. However, the few observed differences were present at a low significance level and for very short periods. These differences in the results obtained were linked to the nature of the test used (selection of a single attribute for TDS and selection of multiple attributes for TCATA).

#### 3.5.2. Differences in Dominance and Citation Duration

One-way ANOVAs were performed to determine the overall flavoring effect on the dominance duration (TDS) of all attributes for each product followed by Tukey tests (Figure S2A). This analysis revealed a negative significant effect of odorants on ODT_red (F = 5.66, *p* = 0.032). No significant effect was observed for ODT_exotic (F = 0.03, *p* = 0.87), ODT_cola (F = 0.25, *p* = 0.62), and ODT_citrus (F = 3.85, *p* = 0.07). For TCATA, the analysis of citation duration also revealed a negative significant effect of flavoring for ODT_red (F = 12.51, *p* = 0.003) (Figure S2B). No effect was observed for ODT_exotic (F = 3.05, *p* = 0.1), ODT_cola (F = 1.69, *p* = 0.21), and ODT_citrus (F = 1.8, *p* = 0.2).

One-way ANOVAs were performed to determine the flavoring impact on dominance or citation duration for each sensory taste attribute (Table 3). For TDS and TCATA, the flavoring effect on dominance or citation duration of the different attributes was very low. ODT_exotic evaluated without a nose-clip had a significantly lower dominance duration for sweetness than when evaluated with a nose-clip (F = 14.34, *p* = 0.002). Relative to ODT_red, the evaluation without a nose-clip had a significantly lower domination and citation duration for acid than the evaluation with a nose-clip (F = 24.64, *p* = 0.001 and F = 27.59, *p* = 0.001, respectively) as well as a significantly lower dominance duration for bitter (F = 5.79, *p* = 0.03). Relative to ODT_citrus evaluated with a nose-clip, ODT_citrus evaluated without a nose-clip had a significantly lower dominance duration for acid (F = 10.40, *p* = 0.006) and for astringent (F = 4.98, *p* = 0.04).

## 4. Discussion

The TDS and TCATA methods were used to study the dynamic sensory perception of liquid, semi-solid, or solid products [17,18,19,20,21,27,28]. However, these two methods have never been used to characterize the sensory properties of ODTs, for which the formulation leads to particular combinations and intensities of sensations not otherwise encountered in foods. This study demonstrated that the sensory perception of ODTs was a dynamic process that spans a longer time than the consumption of current foods. The main objective of this study was to study the dynamic perception of taste and retro-nasal odor to better control and limit off flavors.

These ODTs were characterized by omnipresent flavoring during the entire OFD. However, for both odor and taste attributes, different temporal perception patterns were observed according to the particular dosage form of the ODTs, which could be due to various kinetics of release or sensory interaction effects. ODTs in powder form (exotic and cola) showed a faster decrease in dominance and citation rate of the flavoring attributes than ODTs in tablet form (red and citrus). This can be easily explained by a higher surface available for exchange with the liquid and gaseous phase in the mouth as soon as the beginning of the OFD for the powder ODTs. Concerning the tablets, they were progressively fractionated by mastication and salivation, leading to a slower and longer release and perception due to a more progressive increase in the surface exchange of the ODTs [38].

This aromatic persistence in the ODTs in tablet form (red and citrus) would mask the appearance of some taste sensory perceptions in the second half of the OFD. This phenomenon was not observed for ODT_exotic and ODT_cola. The four products had a common sensory characteristic, a pronounced acidity in the first third of the OFD. This acidity was stable during the OFD for ODT_red and ODT_citrus, unlike ODT_exotic and ODT_cola. It is likely that the dosage form used was responsible for this difference. The tingling and effervescent sensation in the mouth, linked to the use of carbonate in the formulation of the ODTs in tablet form, would prolong the feeling of acidity during tasting [7].

In most cases, we observed a more or less important increase in bitterness in the third part of the mastication time. The logical interpretation would be that bitter compounds were released later in the mouth because of their hydrophobic properties, leading to their slower extraction from the matrix by saliva and a higher persistence of this sensation. However, in the case of ODTs, the main bitter compounds are minerals and vitamins. Taking into account their concentration in the products (Table 1) and their threshold values [10], the bitterness was likely caused by the presence of minerals that are hydrophilic. Thus, they should have been easily extracted in the mouth at the beginning of the OFD. This suggests that in the first two parts of the OFD, the bitterness was masked by sweetness and sourness, which were able to suppress this perception at medium and high intensities [39]. The decrease in sourness and sweetness thus allowed for the bitterness to be more intensely perceived at the end of the OFD. Moreover, this phenomenon could be reinforced by aroma perception potentially enhancing sourness and sweetness according to their taste-association characteristics through cross-modal perceptual interactions [40]. This could be confirmed by further flavor release studies.

The trajectory PCA indicated that the end of product tasting was characterized by an increase in the dominance or citation rate of the bitter attribute. This sensory perception has been linked with the presence of active compounds with bitter potential such as vitamins and minerals [10]. This sensory attribute is generally inversely correlated with the increase in other taste and aromatic attributes, which limit its perception during most of the OFD. Caffeine, exclusively present in ODT_cola, was the direct cause of the strong bitterness perceived by all the panelists during the tasting of this sample [41]. A statistical test was performed to compare the OFD products and revealed a significant difference depending on the dosage form evaluated. More compact than ODTs in powder form, ODTS in tablet form required a longer oral treatment [38], which could be used to better spread the release of off-tastes throughout the OFD, thereby reducing their intensity, on one hand, and making it easier to mask their flavor, on the other hand.

Another component of the study examined the flavoring impact on the sensory perception of taste attributes. It was hypothesized that the flavoring used in the products could boost or attenuate the perception of some taste sensory attributes. Regarding the TDS and TCATA curves of ODT_red and ODT_citrus (with the same formulation but different flavorings), the effects of flavoring on taste sensory perceptions can be suspected. For both methods, ODT_citrus, characterized by the citrus flavoring attribute, was evaluated as slightly more bitter and less sweet than ODT_red, which was flavored with red fruits. Although for TDS, ODT_citrus was significantly more bitter than ODT_red at particular times of tasting, this was not observed for TCATA. This was probably related to the nature of the test (selection of an attribute for TDS and selection of several attributes for TCATA). On the other hand, the nose-clip evaluation and the without nose-clip evaluation of each product did not allow us to conclude a potential effect of flavoring on any taste perception. In the same way, no significant effect of flavoring was observed on dominance and citation duration for ODT_exotic, ODT_cola, and ODT_citrus. One explanation could be that the perception time of sapid compounds is greater than or equal to the perception time of flavoring. It is possible that this observation could be linked to the differences in the release kinetics of the various compounds incorporated in the ODTs studied. The same statistical treatment was applied for each taste sensory attribute. The results, in agreement with the results obtained from the difference curves, were coherent with the interpretations of the results reported above. For both methods, few significant differences were observable. Only the flavoring of ODT_red (red fruits) was at the origin of a significant sensory reduction of the acid attribute probably through cross-modal interactions. As described above, the TDS method highlighted a larger number of significant differences that were probably linked to the nature of the test.

Finally, the relevance of using these temporal analytical methods to characterize these forms of nutritional supplements as well as whether these two methods gave similar and/or complementary information was examined. These two methods were shown to be suitable for achieving the dynamic sensory profile of these nutritional supplements. For both methods, the product curves showed similarities for all products evaluated. The dominant attributes for a product with TDS were also the most cited attributes with TCATA [28]. The two tests allowed for the defining of specific product spaces for the four samples. With a higher F-statistic than that of TDS, TCATA was more discriminating, an observation often observed in other works [24]. The same observation could be said for the PCA trajectories, with a better-marked discrimination of ODT_exotic and ODT_citrus for the TCATA method than for the TDS method.

Although the obtained results were generally similar for both methods, TDS highlighted a greater number of differences in sensory taste attribute perception between the two evaluation conditions. This observation has already been carried out in studies on liquid products [28]. The two methods therefore gave similar and complementary results, the weaknesses of TCATA being corrected by TDS, and conversely. It would be difficult to conclude on the use of one method over the use of the other. To determine the most suitable method, it is important to define the study objectives and the expected results.

This work allowed for the sensory characterization of selected ODTs and to study the flavoring impact on taste sensory perceptions. However, some limitations were highlighted. It turned out that nose-clip notation was a difficult exercise for panelists. In all the studied cases, aroma had no or only a low impact on taste perception. Therefore, to avoid the use of a nose-clip in such tests, the use of ODTs formulated without flavoring would have been more suitable for the panelists to obtain more accurate results. The consideration of texture perception aspects such as mouthcoating, grainy, and, indirectly salivation, would allow for examining texture-taste cross-modal interactions and their potential to lower off tastes. Finally, the other limitation was that the subjects performing the test were not the target population for these ODTs. Indeed, these nutritional supplements are prescribed for people suffering from swallowing disorders. Therefore, an added value to this study should be to perform the same analysis with a representative group of the target population.

## 5. Conclusions

Two temporal sensory analytical methods, TDS and TCATA, allowed for a complete characterization of the taste and flavoring sensory perceptions of ODTs. Both TDS and TCATA highlighted characteristics specific to the two galenic forms studied. These two methods can be used to examine the impact in the formulation on dynamic sensory perceptions. Interactions between tastes were suspected to influence temporal bitterness. However, although some differences could be observed, no major flavoring impact on taste sensory perceptions was found. A more optimized formulation of ODTs, in particular, better use of the sweetness-associated aromas, would make it possible to reduce the use of sweetening molecules and limit off tastes such as bitterness. Another aspect to take into account to improve the formulation of supplements is the flavoring. The main objective of flavoring is to increase the acceptability of ODTs by the users, particularly with the masking of off-flavors. To ensure effective masking of the ODTs, it could be envisaged to use different strategies to modify the release kinetics of the flavoring in the mouth (flavoring encapsulation, use of various matrix). These improvements could contribute to a better pleasant oral experience by providing the improved and prolonged flavoring that patients have come to expect. TDS and TCATA provided similar, but also complementary, information for the sensory characterization of ODTs, as was already shown for everyday consumed foods. Further studies including temporal taste and aroma compound release and temporal sensory experiments with the same formulations of ODTs but with single and multiple omissions of taste or aroma compounds should allow for a better understanding and control of the flavor perception of ODTs.

## Figures and Tables

**Table 1:** Form, composition and flavoring of the four-orodispersible nutritional supplements studied.

	ODT_exotic	ODT_cola	ODT_red	ODT_citrus
**Forms**				
	Powder	Powder	Tablet	Tablet
**Vitamins**				
Vitamin B1	1.1 mg	1.4 mg	5.9 mg	5.9 mg
Vitamin B2	1.4 mg	1.6 mg	7.5 mg	7.5 mg
Vitamin B3	16 mg	18 mg	25 mg	25 mg
Vitamin B5	6 mg	6 mg	11.5 mg	11.5 mg
Vitamin B6	1.4 mg	2 mg	4.11 mg	4.11 mg
Vitamin B8	0.05 mg	0.1 mg	0.075 mg	0.075 mg
Vitamin B9	0.2 mg	0.2 mg	0.2 mg	0.2 mg
Vitamin B12	0.0025 mg	0.001 mg	0.005 mg	0.005 mg
Vitamin C	80 mg	60 mg	250 mg	250 mg
Vitamin D	0.005 mg	X	X	X
Vitamin E	12 mg	X	X	X
Vitamin K	0.025 mg	X	X	X
Vitamin A	0.8 mg	X	X	X
**Minerals**				
Calcium	120 mg	100 mg	50 mg	50 mg
Magnesium	80 mg	100 mg	50 mg	50 mg
Iron	14 mg	X	X	X
Iodine	0.15 mg	X	X	X
Zinc	10 mg	9.5 mg	5 mg	5 mg
Copper	1 mg	X	X	X
Manganese	2 mg	X	X	X
Selenium	0.05 mg	X	X	X
Molybdenum	0.05 mg	X	X	X
**Other substances**				
Caffeine	X	75 mg	X	X
Coenzyme Q10	4.5 mg	X	X	X
**Flavoring**				
	Exotic fruits	Cola	Red fruits	Citrus fruits

**Table 2:** List of attributes, definition and references used for temporal dominance of sensations (TDS) and temporal check-all-that-apply (TCATA).

Modality	Attribute	Definition	Reference
**Taste**	Sweet	Basic taste caused by dilute aqueous solutions of various substances such as sucrose	20 g/L sucrose (Merck 100,892, Merk KGaA, Darmstadt, Germany) in water
	Acid	Basic taste caused by dilute aqueous solutions of various substances such as citric acid	1 g/L citric acid (Merck 100,243, Merk KGaA, Darmstadt, Germany) in water
	Bitter	Basic taste caused by dilute aqueous solutions of various substances such as caffeine	1 g/L caffeine (Sigma-Aldrich 27,602, Sigma-Aldrich Chimie SARL, St. Quentin Fallavier, France) in water
	Salty	Basic taste caused by dilute aqueous solutions of various substances such as sodium chloride	3 g/L sodium chloride (Merck 106,400, Merk KGaA, Darmstadt, Germany) in water
	Metallic	Metallic taste, like that elicited by blood or ferrous sulphate	0.005 g/L ferrous sulphate (Sigma-Aldrich PHR1483, Sigma-Aldrich Chimie SARL, St.Quentin Fallavier, France) in water
**Tactile**	Astringent	Effect of contraction, stretching or puckering of the oral mucosa	0.5 g/L tannic acid (Merck 100,773, Merk KGaA, Darmstadt, Germany) in water
**Retro-nasal aroma**	Citrus fruits	Characteristic aroma of the juice mixture of different citrus fruits (lemon, orange)	Citrus juice, Andros^®^, Biars-Sur-Cere, France
	Red fruits	Characteristic aroma of the juice mixture of different red fruits (strawberry, raspberry)	Strawberry and raspberry juice, Andros^®^, France
	Exotic fruits	Characteristic aroma of the juice mixture of different exotic fruits (mango, orange, pineapple, banana)	Exotic juice, Andros^®^, France
	Cola	Characteristic aroma of certain carbonated drinks such as Coca Cola or Pepsi	Coke, The Coca-Cola Company, Grigny, France

**Table 3:**
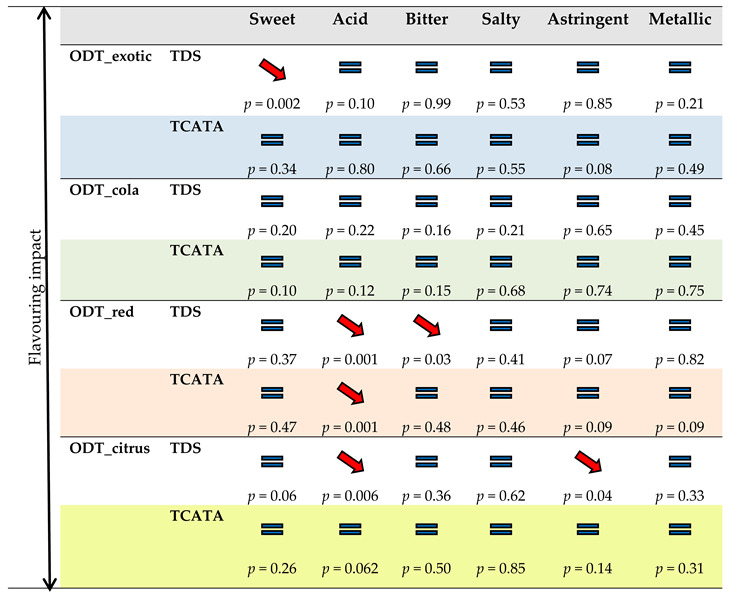
One-way ANOVAs with evaluation type (with nose-clip and without nose-clip) as the independent variable and dominant citation/duration of attributes as the dependent variable to determine the flavoring impact on dominance and citation duration for each product and each attribute. A red downward arrow indicates a significant negative effect at the 95% confidence level. A blue equal sign indicates no significant effect at the 95% confidence level.

## Supplementary Materials

The following are available online at https://www.mdpi.com/2304-8158/9/10/1456/s1, Figure S1: The oral flavor duration (OFD) of the four products evaluated without a nose-clip (WoNC) are depicted on the histograms (A) for TDS and (B) for TCATA. ODT_exotic (blue) and ODT_cola (green) correspond to the ODTs in powder form, while ODT_red (red) and ODT_citrus (orange) are the ODTs in tablet form. F-statistics and associated probabilities are listed below the graph (model: one-way ANOVA; test post hoc: Tukey, alpha = 0.05). The products with a similar OFD are identified by the same letter, Figure S2: The oral flavor duration (OFD) for each product evaluated without a nose-clip (blue, WoNC) and with a nose-clip (green, WNC) are depicted on the histograms (A) for TDS and (B) for TCATA; ns, no significant effect was found at the 95% confidence level; *, a significant positive effect was found at the 95% confidence level; **, a positive effect was found at the 99% confidence level (model: one-way ANOVAs; test post hoc: Tukey, alpha = 0.05).
