# Supplementary material for: Characterizing the Dynamic Taste and Retro-Nasal Aroma Properties of Oral Nutritional Supplements Using Temporal Dominance of Sensation and Temporal Check-All-That-Apply Methods
Source: Foods. 2020 Oct 13;9(10):1456. doi: 10.3390/foods9101456 (PMC7602034; doi:10.3390/foods9101456)
Supplement: Supplementary file 1 [file foods-09-01456-s001.pdf]

Supplementary Materials

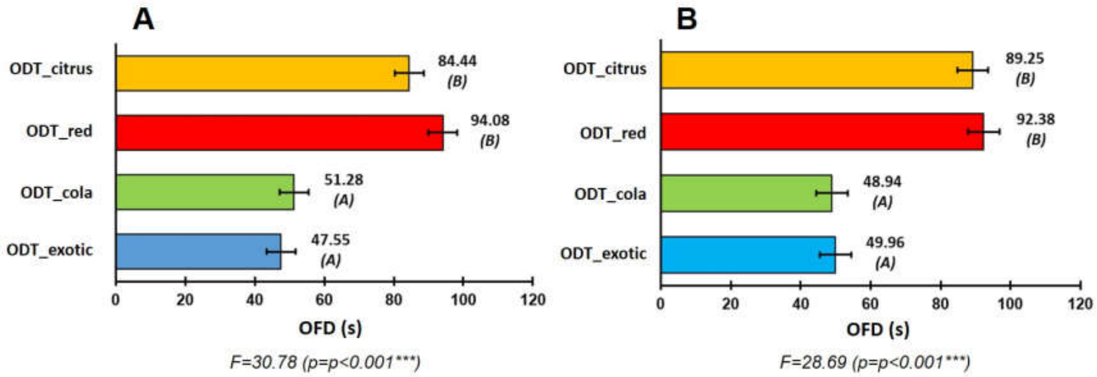

Figure S1. The oral flavor duration (OFD) of the four products evaluated without a nose-clip (WoNC) are depicted on the histograms (**A**) for TDS and (**B**) for TCATA. ODT_exotic (blue) and ODT_cola (green) correspond to the ODTs in powder form, while ODT_red (red) and ODT_citrus (orange) are the ODTs in tablet form. F-statistics and associated probabilities are listed below the graph (model: one-way ANOVA; test post hoc: Tukey, alpha=0.05). The products with a similar OFD are identified by the same letter.

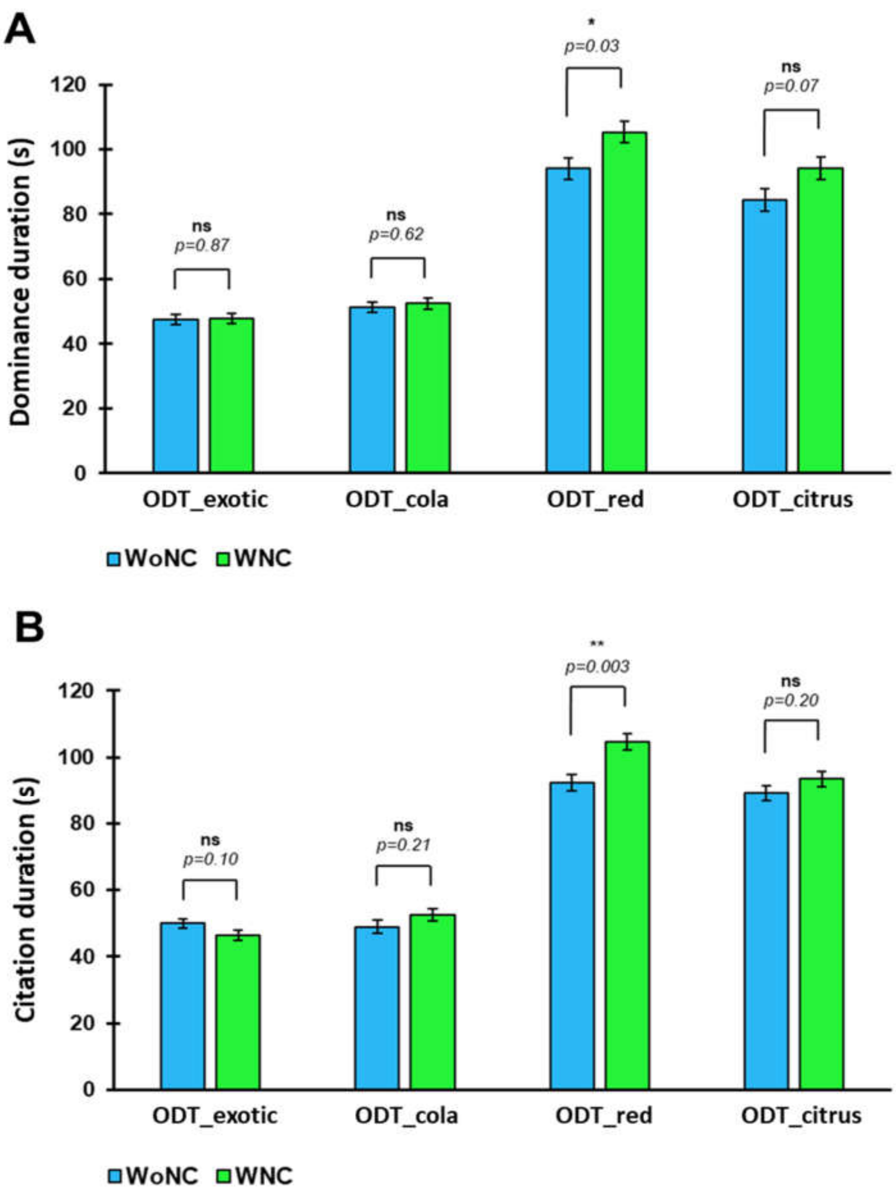

Figure S2. The oral flavor duration (OFD) for each product evaluated without a nose-clip (blue, WoNC) and with a nose-clip (green, WNC) are depicted on the histograms (A) for TDS and (B) for TCATA; ns, no significant effect was found at the 95% confidence level; *, a significant positive effect was found at the 95% confidence level; **, a positive effect was found at the 99% confidence level (model: one-way ANOVAs; test post hoc: Tukey, alpha = 0.05).
